# Novel optical temperature sensor based on emission in Pr^3+^ doped ferroelectric Ba_0.7_Sr_0.3_TiO_3_†

**DOI:** 10.1039/c8ra04228d

**Published:** 2018-07-02

**Authors:** Tang Wei, Ni Haiyong, Zhang Qiuhong, Ding Jianhong

**Affiliations:** Guangdong Research Insitute of Rare-Metal, Guangdong Academy of Science Guangzhou 510650 China t4852065w@126.com

## Abstract

Optical temperature sensing based on the variation of the fluorescence intensity ratio of rare-earth materials has become appealing due to its multiple superiorities over electrical temperature sensing. However, confined by the largest energy separation of two thermally linked levels of rare earth ions, the highest sensitivity of such temperature sensing is essentially smaller than 2878/*T*^2^, as reported previously from diverse systems. In this work, we demonstrate that ultrahigh-sensitive temperature sensing can be achieved from Pr^3+^-doped (Ba_0.7_Sr_0.3_)TiO_3_ based on the intensity ratio of the ^1^D_2_–^3^H_4_ emission to the ^3^P_0_–^3^H_4_ emission. The ratio can be increased as much as 90-fold when the temperature rises from room temperature to 513 K, nicely fitting a thermally linked-levels like equation and showing an ultrahigh sensitivity of 4275.1/*T*^2^. The striking change of the ratio is attributed to the interaction between the two emission levels and the intervalence charge transfer state. This work may have provided a distinct route in the field of optical temperature sensing utilizing rare-earth-doped materials. In addition, the resultant product also possesses excellent photoluminescence and ferroelectric properties, showing promising potentials in multifunctional devices for practical applications.

## Abstract 2

Optical temperature sensing based on the variation of the fluorescence intensity ratio of rare-earth materials has become appealing due to its multiple superiorities over electrical temperature sensing.

## Graphical abstract

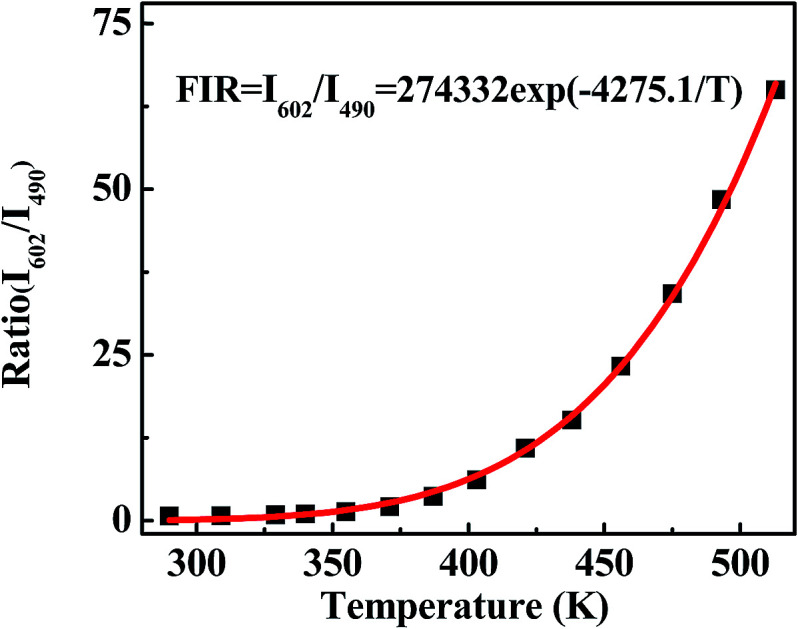

## Introduction

Currently, the optical thermometric technique based on the fluorescence intensity ratio (FIR) in a phosphor material is attracting intensive investigations, for its advantages such as non-contact, rapid response, and high spatial/temperature resolutions that facilitate temperature detecting for harsh environments or fast moving objects.^1–7^ Most of the previous thermometric material studies focus on the thermally coupled level-pairs (TCL) of rare earth ions (for example, ^2^H_11/2_, ^4^S_3/2_ levels of Er^3+^; ^3^F_2,3_, ^3^H_4_ levels of Tm^3+^; and ^4^F_7/2_, ^4^F_3/2_ levels of Nd^3+^).^8–13^ In a definite TCL phosphor, population at the upper and lower levels of TCL would change oppositely with increasing temperature, inducing variation in the FIR of these two levels. For this type of thermometric material, to avoid exceedingly low upper-level populations, it is widely accepted that the energy separation of the two thermally linked levels of rare-earth ions is less than 2000 cm^−1^,^14–16^ which essentially confines the highest value of *S* to no larger than 2878/*T*^2^. Indeed, all the reported *S* values from diverse rare earth ions, *e.g.*, Tm^3+^, Nd^3+^, Eu^3+^, Dy^3+^, Ho^3+^, and Er^3+^, are smaller or comparable to this value.^11,12,16–20^ The limited *S* is apparently a big obstacle for the further promotion and practical application of optical sensors based on the fluorescence intensity ratio of rare-earth materials.

Recently, besides searching for excellent TCL thermometric materials, great efforts have also been made to develop a new thermometric strategy. For example, the phonon assisted energy transfer between Tb^3+^ and Eu^3+^ ions has been employed in optical thermometry.^1,5,21^ This thermometric strategy provides better signal discriminability, but is only applicable at a temperature below 320 K. Quantum dots decorated by lanthanide–organic complexes have also been applied as thermometric materials relying on the different thermal quenching behaviors between quantum dots and lanthanide ions. However, the FIR of these quantum dots is easily influenced by the other environment parameters such as acidity and alkalinity, which would introduce errors in practical temperature detection.^22,23^

It is known that some lanthanide ions (Pr^3+^ or Tb^3+^) and the d^0^ electron configured transition metal ions (Mn^+^ = Ti^4+^, V^5+^, Nb^5+^, Ta^5+^, Mo^6+^ or W^6+^) in an oxide host could form the so-called metal-to-metal intervalence charge transfer (IVCT) state, which would interfere in emission of the f levels in the lanthanide ions, and therefore introduce a high temperature-dependence for the luminescence.^24–26^ Meanwhile, in our previous work, we demonstrated that the IVCT mechanism plays a key role in Pr^3+^-doped (K_0.5_Na_0.5_)NbO_3_ optical oxygen sensing; the decreased energy position of the IVCT state with the increased oxygen concentration induced the enhanced ^1^D_2_–^3^H_4_ emission and decreased ^3^P_0_–^3^H_4_ emission, leading to the large oxygen sensing response of both the absolute ^1^D_2_–^3^H_4_ emission intensity and the ^1^D_2_–^3^H_4_/^3^P_0_–^3^H_4_ intensity ratio.^27^ Based on these researches, a novel temperature sensing strategy that utilizes the IVCT state interfered Pr^3+^ luminescence to perform optical thermometry is proposed.

As an important member of ferroelectric materials, studies of (Ba, Sr)TiO_3_ materials have been a widely welcomed topic due to their high permittivity, low dielectric loss, high tunability coefficient, quick reaction velocity, anti-breakdown ability and simple fabrication process, *etc.* Meanwhile, among many advantages is the fact that the (Ba, Sr)TiO_3_ family is lead-free, and therefore compliant with nowadays requirements for environmentally benign materials.^28^ Moreover, (Ba, Sr)TiO_3_ contains one kind of IVCT (*i.e.*, Pr^3+^–Ti^4+^ IVCT). In this work, we studied the photoluminescence and ferroelectric properties of Pr^3+^ doped Ba_0.7_Sr_0.3_TiO_3_. The temperature sensor dependence on the fluorescence intensity ratio of the traditional thermally linked ^3^P_1_–^3^H_5_ and the ^3^P_0_–^3^H_5_ emissions has been studied, which sensitivity is not that significant.^29,30^ Remarkably, FIR of the Pr^3+ 3^P_0_ and ^1^D_2_ emissions exhibits high temperature dependence, the intensity ratio of the ^1^D_2_–^3^H_4_ emission to the ^3^P_0_–^3^H_4_ emission at 513 K can be increased to as high as 90-fold of that at room temperature, and this temperature-dependent ratio can be nicely fitted by a thermally linked-levels-like equation, strikingly showing an ultrahigh *S* of 4271.1/*T*^2^, which is about 150% of the upper limit of *S* as introduced above. The configurational coordinate diagram is applied to analyze the mechanism of the temperature-dependent luminescent characteristics, and thermo-induced relaxation between the Pr^3+ 3^P_0_ and ^1^D_2_ levels through the IVCT state is further demonstrated to be the primary cause for the temperature sensing performance of Pr^3+^-doped Ba_0.7_Sr_0.3_TiO_3_. Meanwhile, it is revealed that doping with Pr^3+^ can further promote the ferroelectric performance of Ba_0.7_Sr_0.3_TiO_3_. Integrating with the excellent PL and ferroelectric properties, the ultra-high sensitivity on temperature sensing of Pr^3+^-doped Ba_0.7_Sr_0.3_TiO_3_ not only indicates its remarkable potentials in multifunctional devices, but also may have opened up a distinct and fresh route in the field of highly sensitive optical temperature sensing utilizing rare-earth-doped materials.

## Experimental

Ba_0.7_Sr_0.3_TiO_3_ (BST) and Ba_0.7_Sr_0.295_Pr_0.005_TiO_3_ (BST:Pr^3+^) nanoparticles were synthesized by a hydrothermal method. Firstly, 40 ml aqueous solution of strontium nitrates and barium nitrates (1.119375 mol L^−1^, 99%, Aladdin) and praseodymium nitrates (0.005625 mol L^−1^, 99%, Aladdin) were mixed with 23 ml ethanol solution of tetrabutyl titanate (1.25 mol L^−1^, 99%, Aladdin). The pH value of the mixed solution was adjusted to be 13.5 by adding NaOH. The reactive solution was then sealed in a Teflon autoclave at 200 °C for 48 h. After cooling down to room temperature, the obtained product was thoroughly washed by deionized water and ethanol, eventually dried overnight in air.

The crystallization nature and morphology of the samples were characterized using powder X-ray diffraction (Rigaku D/MAX-2600/PC with Cu Kα radiation) and scanning electron microscopy (SEM; JEOL 6700F). Photoluminescence (PL) and photoluminescence excitation (PLE) spectra of the samples were measured using a spectrofluorometer (HORIBA, Fluoromax-4). The polarization *vs.* electric field (*P*–*E*) hysteresis loop was obtained at 50 Hz using a Precision Premier II tester (Radiant Technology USA) at room temperature (30 kV cm^−1^).

## Results and discussion

**Fig. 1:**
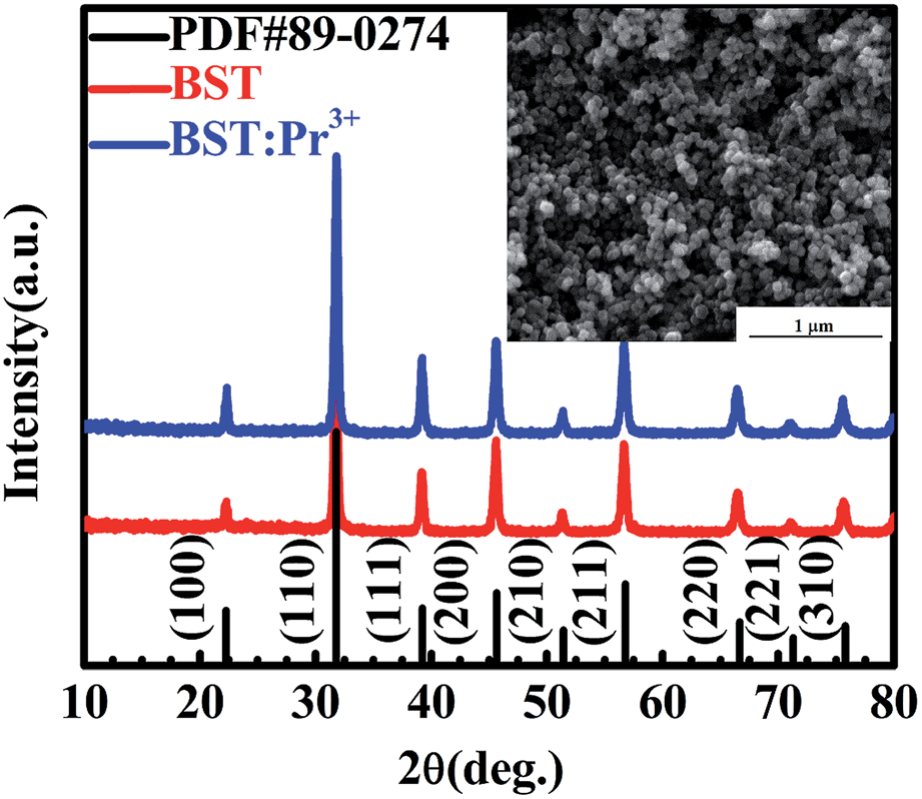
XRD patterns of BST and BST:Pr^3+^ nanoparticles. SEM image of the BST:Pr^3+^ nanoparticles is presented in the inset.

Fig. 1 present XRD patterns of BST and BST:Pr^3+^ samples. All the peaks in the spectra can be assigned to the BST host structure (PDF#89-0274), and no phases related to impurities were observed. A SEM image of the as-prepared BST:Pr^3+^ sample is shown in the inset of Fig. 1. The average size of these particles is about 60 nm. All these nanoparticles of BST and BST:Pr^3+^ samples appear quasi-spherical morphology with a similar size.

**Fig. 2:**
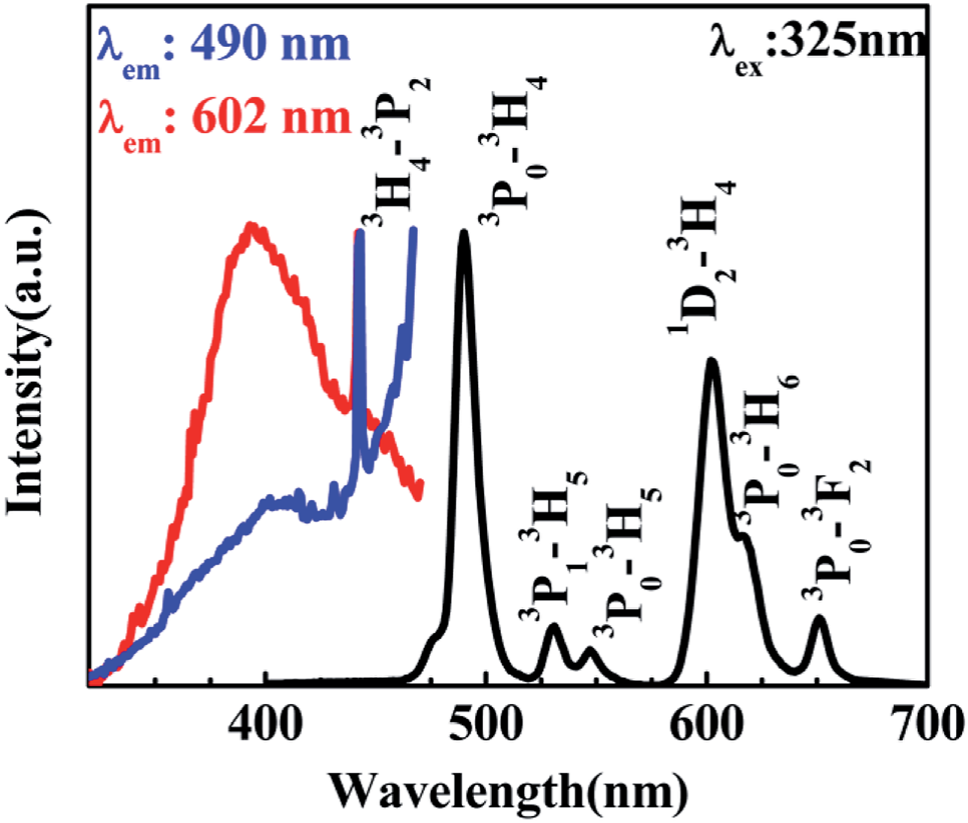
PL spectrum of the BST:Pr^3+^ nanoparticles with an excitation of 325 nm and its PLE spectra monitored at 490 nm and 602 nm.

According to the PL of BST and BST:Pr^3+^ (ESI Fig. S1†). The intrinsic BST almost have no PL properties relative to BST:Pr^3+^, it can be inferred that the PL of BST:Pr^3+^ is derived from Pr^3+^. Fig. 2 shows PL and PLE spectra of BST:Pr^3+^ samples normalized by their maximum intensity. Under 325 nm excitation, the PL spectrum showed a strong blue-green emission at 490 nm (^3^P_0_–^3^H_4_), a strong red emission at 602 nm (^1^D_2_–^3^H_4_), together with two weak green emissions at 530 nm (^3^P_1_–^3^H_5_) and 547 nm (^3^P_0_–^3^H_5_) and two weak red emissions at 617 nm (^3^P_0_–^3^H_6_) and 651 nm (^3^P_0_–^3^F_2_). The PLE spectra monitored at 490 nm and 602 nm were exhibit a broad bands centered at 392 nm, which can be attributed to the BST host absorption and the IVCT absorption.^31,32^ Besides, the sharp peak at 450 nm is ascribed to ^3^H_4_ → ^3^P_2_ transition of Pr^3+^.

**Fig. 3:**
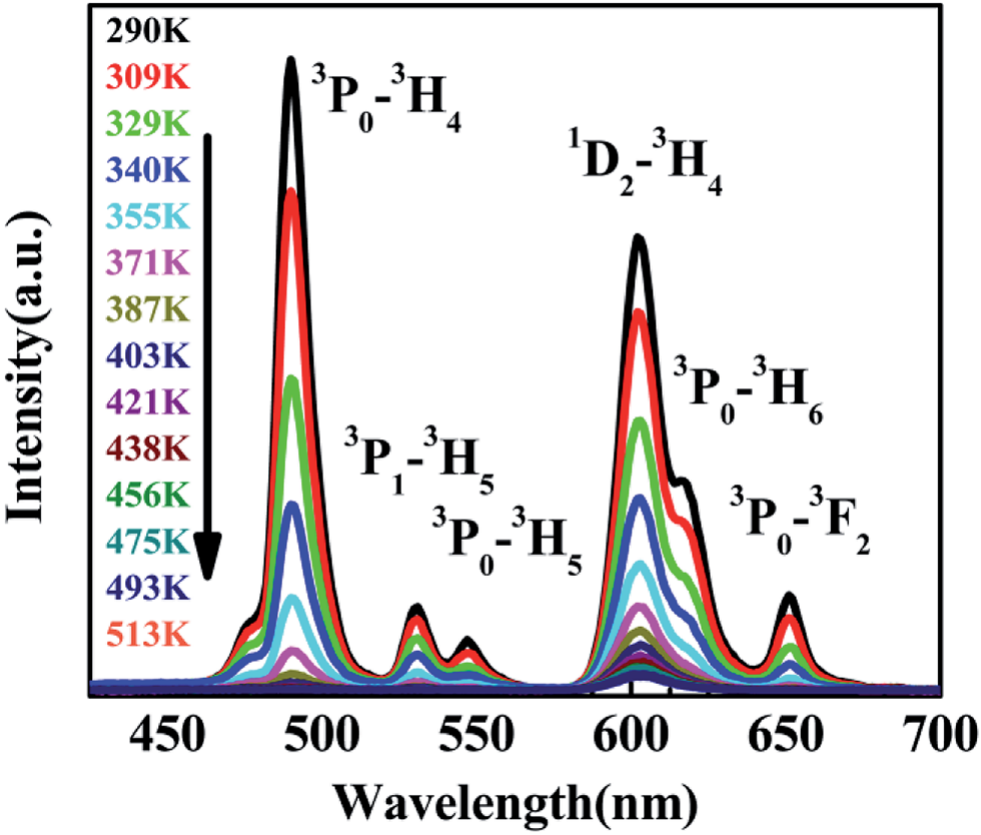
PL of BST:Pr^3+^ nanoparticles at different temperatures under 325 nm excitation.

**Fig. 4:**
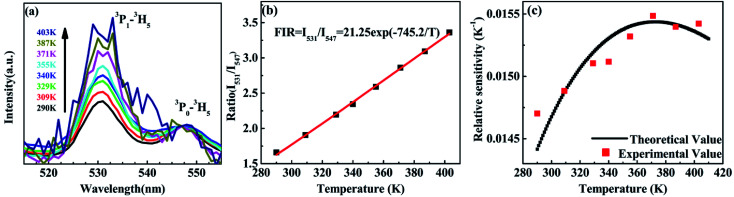
(a) PL of the BST:Pr^3+^ nanoparticles at different temperature (normalized by the ^3^P_0_–^3^H_5_ emission intensity); (b) the intensity ratio of the ^3^P_1_–^3^H_5_ emission to the ^3^P_0_–^3^H_5_ emission as a function of temperature in the range of 293–403 K; (c) relative sensitivity as a function of temperature in the range of 290–403 K.

The variation of emissions of BST:Pr^3+^ with the temperature over the 290 to 513 K is depicted in Fig. 3, it can be observed that the peak positions of these emission bands are hardly changed and the absolute intensities decrease gradually with temperature. The relative emission intensities for 531 and 547 nm, 490 and 602 nm emissions respond differently to the change of temperature. The intensity of two peaks corresponding to 531 nm (^3^P_1_–^3^H_5_) and 547 nm (^3^P_0_–^3^H_5_) transitions at different temperatures are shown in Fig. 4(a). It is obvious from Fig. 4(a) that the intensity of the peak ^3^P_1_–^3^H_5_ decreases more rapidly compared to ^3^P_0_–^3^H_5_ due to the thermal agitation. The ^3^P_1_ and ^3^P_0_ states of Pr^3+^ are closely spaced with a separation of about 550 cm^−1^, which belong to TCL.^33^ As a result, the FIR for the emissions from TCL of Pr^3+^ ions can be described as:^34,35^1
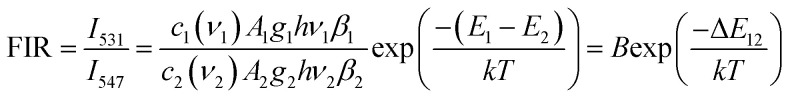
where the emission intensities for the 531 and 547 nm are *I*_531_ and *I*_547_, respectively. The values of *c*_1_(*ν*_1_) are related to the response of the detection system. *A*_1_, *g*_1_, *hν*_1_ and *β*_1_ are the spontaneous radiative rates, the degeneracy, the photon energy and the branching ratio, respectively, for transitions from the excited ^3^P_1_ and ^3^P_0_ levels to ^3^H_5_ state. *B* is a constant; Δ*E*_12_ is the effective energy difference between the ^3^P_1_ to ^3^P_0_ state; *k* is the Boltzmann constant; and *T* is the absolute temperature.

The temperature dependence of these emissions at 531 and 547 nm in the range of 290–403 K (Fig. 4(b)) show clear rise in FIR value with temperature, reaching a maximum value when the temperature approaches the maximal experiment temperature 403 K. From a curve fitting of the experimental data, the fitted constants *B* and Δ*E*_12_ are 21.25 and 518 cm^−1^. The fitted Δ*E*_1_ is close to the experimental value 550 cm^−1^. To further understand the temperature response of the BST:Pr^3+^, it is important to investigate the sensing sensitivity, this can be defined from:^34^2
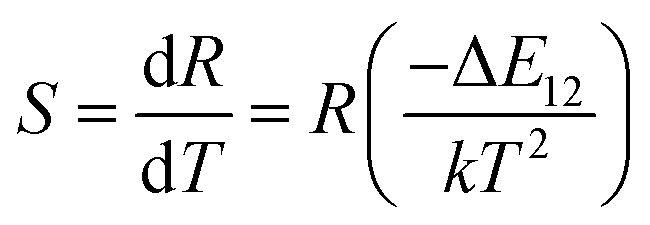


The sensitivity as a function of temperature (Fig. 4(c)) is 745.2/*T*^2^ and reach its maximum value of 0.015 K^−1^ at 375 K in the temperature range of interest, this phenomenon consist with other groups.^20,28^ It can found that, the PL spectrum of 531 nm and 547 nm emissions is overlap, the precise intensity reading is not very convenient. And due to the rather small energy separation of the ^3^P_1_ to ^3^P_0_ state, only *S* as low as 745.2/*T*^2^ was obtained. So the thermally linked ^3^P_1_–^3^H_5_ and the ^3^P_0_–^3^H_5_ emissions are not suitable for the modern development of high performance temperature detection.

**Fig. 5:**
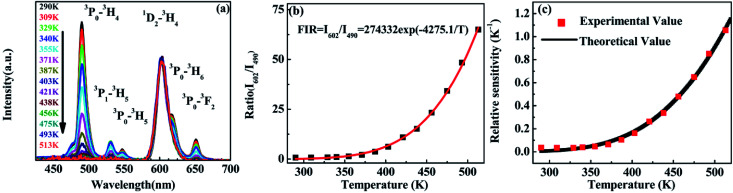
(a) Emissions of BST:Pr^3+^ nanoparticles at different temperatures which normalized at 602 nm. (b) Ratio of emissions between 490 and 602 nm as a function of temperature in the range of 290–513 K. (c) Relative sensitivity as a function of temperature in the range of 290–513 K.

Revealed by PL spectra normalized by the ^1^D_2_–^3^H_4_ emission intensity (refer to Fig. 5(a)), the intensity of all the ^3^P_0_-related emissions was reduced much faster than that of the ^1^D_2_–^3^H_4_ emission upon the temperature rise. Fig. 5(b) presents the calculated intensity ratio of the ^1^D_2_–^3^H_4_ emission to the ^3^P_0_–^3^H_4_ emission by referring the maximum emission intensity at 602 nm and 490 nm in the PL spectra, as a function of temperature in the range of 293–513 K. Excitingly, a huge increase as high as 90 fold of the ratio was achieved when the temperature increased from room temperature to 513 K. The intensity ratio can be fit nicely with a thermally linked-levels-like equation, deducing an ultrahigh sensitivity of 4275.1/*T*^2^, which is not only ∼6 times higher than that of the optical sensors based on thermally linked ^3^P_1_ and ^3^P_0_ levels of Pr^3+^, but also much higher than all the reported optical sensors based on the fluorescence intensity ratio of rare-earth materials^8,9,13,29,34,39,40^ (as listed in Table 1), where the highest sensitivity of 2878/*T*^2^ was anticipated. The sensitivity keeps increasing in our experimental temperature range and reaches the maximum value 1.055 K^−1^ at 513 K as shown in Fig. 5(c).

**Table 1:** Summarized temperature sensing performance of rare-earth ions-doped materials

Rare-earth ion (host)	Transition	Sensitivity (K^−1^)	Temperature range (K)	Ref.
Pr^3+^ (BaSrTiO_3_)	^3^P_0_, ^1^D_2_ → ^3^H_4_	4275.1/*T*^2^	290–513	This work
Pr^3+^ (BaSrTiO_3_)	^3^P_1_, ^3^P_0_ → ^3^H_5_	745.2/*T*^2^	290–403	This work
Er^3+^ (BaTiO_3_)	^2^H_11/2_, ^4^S_3/2_ → ^4^I_15/2_	1026.8/*T*^2^	294–923	8
Nd^3+^ (oxyfluoride glass ceramic)	^4^F_7/2_, ^4^F_3/2_ → ^4^I_9/2_	3010.1/*T*^2^	303–623	9
Tm^3+^ (NaYbF_4_)	^3^F_2_,_3_, ^3^H_4_ → ^3^H_6_	2007.0/*T*^2^	323–773	13
Pr^3+^ (tellurite glass)	^3^P_1_, ^3^P_0_ → ^3^H_5_	879.9/*T*^2^	293–473	29
Pr^3+^ (β-NaYF_4_)	^3^P_1_, ^3^P_0_ → ^3^H_5_	675.7/*T*^2^	120–300	34
Dy^3+^ (Y_4_Al_2_O_9_ crystal)	^4^I_15/2_, ^4^F_9/2_ → ^6^H_13/2_	1709.4/*T*^2^	296–973	39
Ho^3+^ (BaTiO_3_-(Na_0.5_Ho_0.5_)TiO_3_)	^5^F_4_, ^5^S_2_ → ^5^I_8_	933.8/*T*^2^	80–600	40

**Fig. 6:**
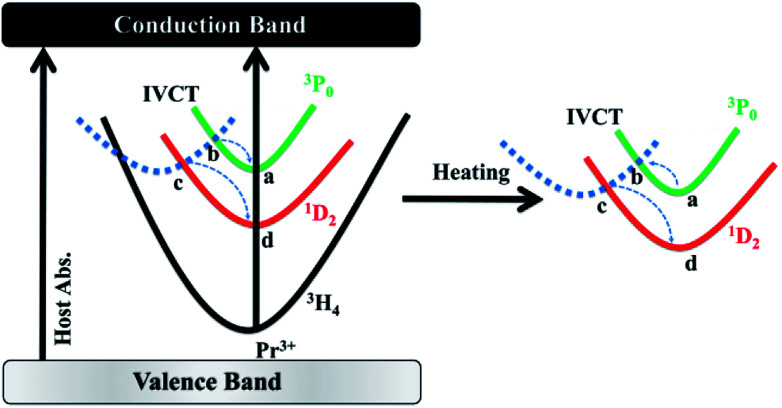
Illustration of the temperature sensing mechanisms of the BST:Pr^3+^ nanoparticles.

Given that the energy separation between ^3^P_0_ and ^1^D_2_ (∼4000 cm^−1^) is apparently too large to promote the multiphonon relaxation between the two states, a new viewing angle is, therefore, required to understand the intensity ratio of the two emissions. Interestingly, it has been suggested that, there is a host-dependent IVCT state in Pr^3+^ doped titanates.^36–38^ In our present work, we demonstrate the IVCT state can interfere with the excited states of Pr^3+^ including the ^3^P_0_ level and the ^1^D_2_ level, and induce the reduction of the Pr^3+^ emissions or/and provide a de-excitation pathway from the ^3^P_0_ level to the ^1^D_2_ state. For a clear illustration for this distinct phenomenon, we depict interactions between the IVCT state and Pr^3+^ ions in Fig. 6. Under 325 nm excitation, the electron in the valance band is transferred to the conduction band. After fast non-radiative relaxation, the electrons in conduction band relax to the IVCT and the ^3^P_0_ states simultaneously with different non-radiative rates. The electrons in IVCT state returns to the minimum potential energy position and rapidly transfers to the ^3^P_0_ and ^1^D_2_ state with the assistance of thermal phonons. Then we can obtain the blue and red light under 325 nm excitation as shown in Fig. 2. With the rising of temperature, electrons in ^3^P_0_ state not only transfer to ^3^H_4_ state, but also transfer to IVCT state by absorbs thermal energy. Then, these electrons in IVCT state relax to ^1^D_2_ level, which induce the electrons in ^1^D_2_ state increases relative to ^3^P_0_ state with the temperature raises, and the intensity ratio of ^1^D_2_–^3^H_4_/^3^P_0_–^3^H_4_ increases constantly. From the above, it can conclude that thermo-induced relaxation from the ^3^P_0_ to ^1^D_2_ level through the IVCT state is the primary cause for the high temperature-dependent FIR in BST:Pr^3+^. Although the mechanism deserves further investigation, the ultrahigh sensitivity based on the ratio of ^1^D_2_–^3^H_4_ and ^3^P_0_–^3^H_4_ emissions may have provided a fresh and favourable route for optical temperature sensing.

**Fig. 7:**
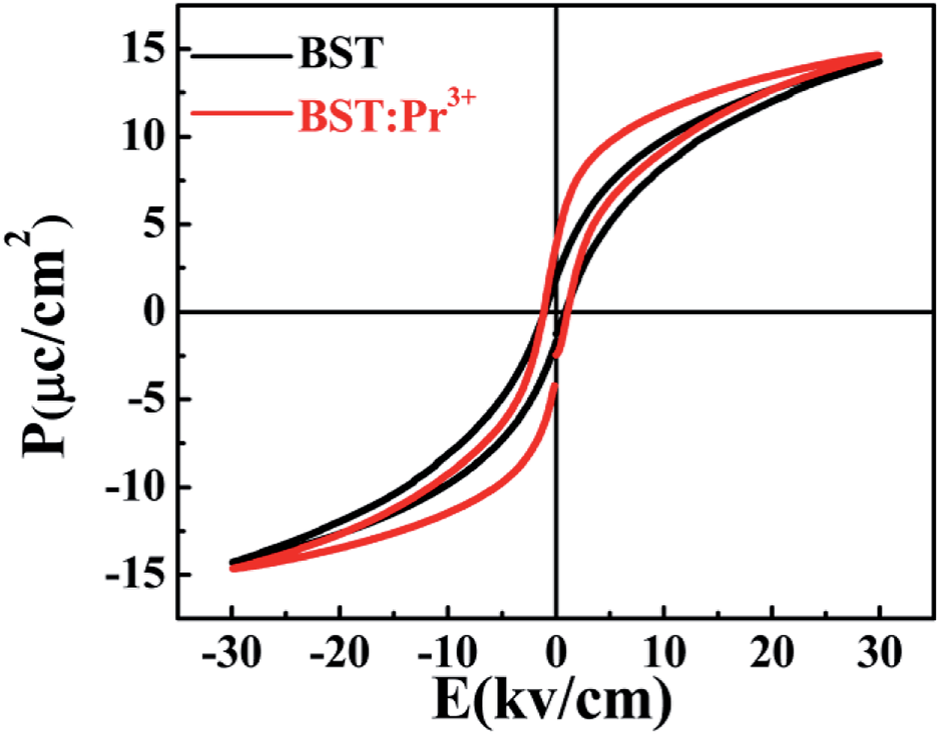
Polarization–electric field hysteresis loops of the BST and BST:Pr^3+^ samples.

Fig. 7 shows the ferroelectric hysteresis loops of BST and BST:Pr^3+^ nanoparticles, measured at room temperature. The remnant polarization of the samples changed from 1.89 to 3.79 μC cm^−2^ when Pr^3+^ doped. The improved Pr in the BST:Pr^3+^ may be due to the Pr^3+^ substitution can increase the number of switchable domain by applied field. With the trivalent Pr^3+^ unequivalently substituting the univalent (Ba_0.7_Sr_0.3_)^2+^, to maintain charge neutrality A-sites ionic vacancies will occur in the BST and form defect dipole with Pr^3+^. In general, the electric performances of the BST depend not only on the structure of the domain and the movement of non-180 °C domain walls but also on the effect of the defect structure on the domains configuration and the interaction of between the defects and domain walls. During the testing process, the stress in the domain walls would be released by migrating to the defects, which would promote the domain walls to move laterally, leading to the reorientation and growth of the domains. And that a larger number of domains contribute to Pr for BST:Pr^3+^.^41^ The coercive electric field (*E*_c_) of BST and BST:0.5%Pr^3+^ are 0.84, 1.01 kV cm^−1^, respectively. When Pr^3+^ substitutes the (Ba_0.7_Sr_0.3_)^2+^, the lattice distortion is induced by the different ionic radius. And the large ion migration results in large polarization, which leads the *E*_c_ of BST:0.5%Pr^3+^ is larger than BST. This results conformity with other works.^42–44^ The enhancement of ferroelectric and the excellent PL performance in BST:Pr^3+^ nanoparticles, makes it possible in applications of the ferroelectric/luminescent multifunctional devices.

## Conclusions

In summary, BST:Pr^3+^ ferroelectric samples were prepared by a hydrothermal method. The green emissions at 531 nm and 547 nm were investigated under 325 nm optical excitation in a temperature range from 290 K to 403 K. This investigation revealed that the value of the FIR for *I*_531_/*I*_547_ increases gradually with increasing temperature, and a maximum sensitivity for the BST:Pr^3+^ ceramic of 0.015 K^−1^ at 375 K was reached. Furthermore, BST:Pr^3+^ operated a high sensitivity optical temperature sensor based on *I*_490_/*I*_602_ with a temperature range of 290–513 K, which could reach the maximum value 1.055 K^−1^ at 513 K. Instead of multiphonon relaxation between two thermally coupled states, the big variation of the intensity ratio of the ^1^D_2_–^3^H_4_ emission to the ^3^P_0_–^3^H_4_ emission upon temperature rise is closely relevant to their interaction with the IVCT state. The ultrahigh sensitivity based on the two emissions may shed a bright light on the promotion of optical temperature sensing and understanding of the involved mechanism. Moreover, the ferroelectric exhibits excellent ferroelectric properties.

## Conflicts of interest

There are no conflicts to declare.

## Supplementary Material

RA-008-C8RA04228D-s001
