# Supplementary material for: Novel optical temperature sensor based on emission in Pr3+ doped ferroelectric Ba0.7Sr0.3TiO3
Source: RSC Adv. 2018 Jul 2;8(42):23996–4001. doi: 10.1039/c8ra04228d (PMC9081777; doi:10.1039/c8ra04228d)
Supplement: RA-008-C8RA04228D-s001 [file RA-008-C8RA04228D-s001.pdf]

Electronic Supplementary Material (ESI) for RSC Advances.
This journal is © The Royal Society of Chemistry 2018

Supplementary Information

Novel optical temperature sensor based on emission in Pr^{3+} doped ferroelectric $Ba_{0.7}Sr_{0.3}TiO_3$

Tang Wei,* Ni Haiyong, Zhang Qiuhong and Ding Jianhong

Guangdong Research Insitute of Rare-Metal, Guangdong Academy of Science, Guangzhou 510650,China, Email:t4852065w@126.com.

* Correspondence should be addressed to t4852065w@126.com

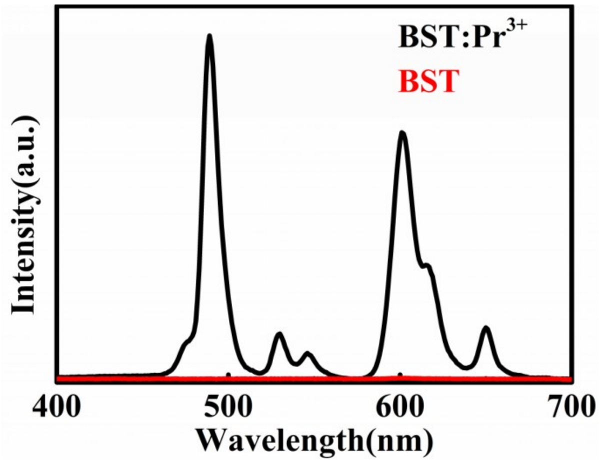

Supplementary Fig.S1 PL of BST and BST:Pr^{3+}

Fig. S1 shows the intrinsic BST almost have no PL properties relative to BST:Pr^{3+}. It can be inferred that the PL of BST:Pr^{3+} is derived from Pr^{3+}.
